# The Role of Purine Metabolism and Uric Acid in Postnatal Neurologic Development

**DOI:** 10.3390/molecules30040839

**Published:** 2025-02-11

**Authors:** Lauren N. Mileti, James D. Baleja

**Affiliations:** Master’s Program in Biomedical Sciences, Departments of Medical Education and Developmental, Molecular, and Chemical Biology, Tufts University School of Medicine, 136 Harrison Avenue, Boston, MA 02111, USA; lauren.mileti@tufts.edu

**Keywords:** purine, uric acid, brain development, substantia nigra pars compacta, Lesch–Nyhan disease

## Abstract

This review explores the essential roles of purine metabolism including the catabolic product, uric acid, in the development of dopaminergic neurons of the substantia nigra pars compacta. The high energy requirements of the substantia nigra pars compacta alongside necessary purinergic neurotransmission and the influence of oxidative stress during development makes these neurons uniquely susceptible to changes in purine metabolism. Uric acid’s role as a central nervous system antioxidant may help to ameliorate these effects in utero. Understanding the mechanisms by which purines and uric acid influence development of the substantia nigra pars compacta can help further explain neurologic consequences of inborn errors of purine metabolism, such as Lesch–Nyhan disease.

## 1. Introduction

Several diseases caused by inborn errors of purine metabolism result in neurologic symptoms that are poorly understood. Purines and purine metabolism perform complex but essential roles in neurologic development, particularly effecting the dopaminergic neurons of the substantia nigra pars compacta (SNc). SNc neurons have exceptionally high energy demands compared to the rest of the brain, such as the dopaminergic neurons of the ventral tegmental area [1,2]. This high energy requirement necessitates a significant need for purines. Purines also serve other functions in the SNc, including extracellular neurotransmission and intracellular signaling [3,4]. The influence of purine metabolism on developmental pathways in the SNc warrants further investigation to provide insight into neurological disease and potential therapeutics for patients.

Purines are heterocyclic compounds composed of a pyrimidine and an imidazole ring that play crucial roles in several fundamental biological processes. The two principal purines are adenosine and guanine, which are vital to cellular function. Both purines are integral to the synthesis of nucleotides, which are required for the replication and repair of DNA and RNA, processes that are especially critical during periods of rapid growth and development [5].

In addition to their roles in nucleotide synthesis, adenosine and guanine are involved in intracellular signaling. Adenosine is a precursor to cyclic AMP (cAMP), a secondary messenger in the protein kinase A (PKA) pathway [6]. This pathway includes adenylyl cyclase, an enzyme that catalyzes the conversion of ATP to cAMP. cAMP activates PKA, which regulates various downstream targets involved in gene expression, metabolism, and synaptic plasticity. Guanine, as a component of GTP, is central to G-protein-coupled receptor (GPCR) signaling, which regulates a wide array of cellular processes, including neurotransmission and synaptic plasticity [7,8,9].

Understanding the role of the de novo and salvage pathways is essential in the context of neurologic development. Both embryonic and postnatal development require a supply of purines to support neurogenesis, synaptogenesis, and myelination. Disruptions in either pathway can cause significant biochemical imbalances leading to anatomical and cellular changes [10].

Purines are synthesized from two pathways: the de novo synthesis pathway and the salvage pathway. The de novo synthesis of purines is a highly conserved, energetically intensive process that involves the formation of the purinosome—a complex containing ten or more enzymes that synthesizes purines [11]. In addition to purinosome formation, the filamentation of other enzymes, including inosine 5′-monophosphate dehydrogenase (IMPDH), further regulates synthesis [12,13]. The accumulation of IMPDH nuclear inclusion bodies correlates with normal aging of the substantia nigra pars compacta [14].

**Figure 1:**
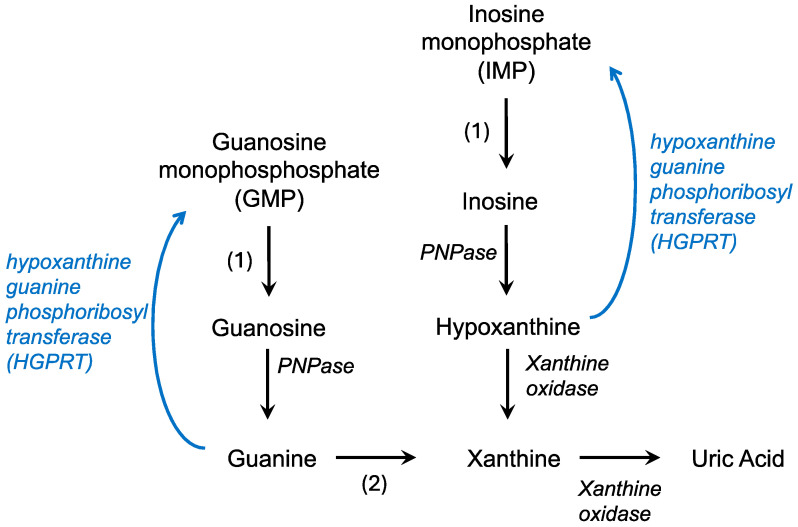
Purine catabolism and salvage pathways. Degradation pathways are in black while the salvage pathway is in blue. PNPase—polynucleotide phosphorylase; 1—nucleotidase; 2—guanine deaminase.

The de novo synthesis of purines builds on a phosphoribosyl pyrophosphate (PRPP) backbone, derived from the pentose phosphate pathway, and ultimately forms IMP while utilizing CO_2_, amino acids, and tetrahydrofolate [15]. IMP can then react with either aspartate or glutamine to form AMP or GMP, respectively. In contrast, the salvage pathway recycles free purine bases and nucleosides derived from the breakdown of nucleic acids (Figure 1). Hypoxanthine, a purine derivative, and guanine are substrates for hypoxanthine-guanine phosphoribosyl transferase (HGPRT or HPRT) which combines these molecules with PRPP to form IMP or GMP, respectively. Adenine phosphoribosyltransferase catalyzes a similar reaction, converting adenine to AMP. This recycling is less energy intensive, utilizing the hydrolysis of only one ATP or GTP compared to the five used in the de novo pathway to efficiently maintain purine levels in the cell [4].

The regulation of these pathways involves allosteric feedback towards PRPP synthetase with AMP, ADP, and GDP, indicating low cellular energy stores, and inhibiting the formation of PRPP to shunt resources towards glycolysis and cellular respiration. The committed step of de novo synthesis quickly follows; the combination of PRPP, glutamine, and water produces 5-phosphoribosylamine, glutamate, and pyrophosphate via glutamine PRPP aminotransferase. Glutamine PRPP aminotransferase is feed-forward activated by PRPP and inhibited by the end products (AMP, GMP, IMP) [4].

In the body, purine catabolism proceeds primarily in the liver. AMP (adenosine monophosphate) is converted to IMP (inosine monophosphate) and eventually to hypoxanthine by purine nucleoside phosphorylase (Figure 1). Hypoxanthine is converted to xanthine by xanthine oxidase [16]. Guanine degradation converges on the formation of xanthine. Xanthine oxidase also catalyzes the conversion of hypoxanthine to uric acid. In contrast to humans, in most fish, amphibians, and mammals, uric acid is broken down into allantoin by an enzyme called uricase [17]. Uric acid is the final product of purine catabolism in animals that lack the enzyme uricase, including hominids, great apes, and some bird species [18].

Disruptions in purine metabolism pathways can lead to a range of neurological disorders, as seen in diseases such as Lesch–Nyhan syndrome, which is characterized by HGPRT deficiency. This deficiency impairs purine recycling, resulting in excessive uric acid production and severe neurodevelopmental consequences. The dopaminergic neurons of the SNc, with their high purine demands, are particularly vulnerable to metabolic disturbances, further contributing to motor dysfunction and cognitive impairment. Investigating the relationship between purine metabolism, neuronal energy requirements, and neurotransmission in the SNc can provide key insights into the pathophysiology of purine-related disorders and inform the development of targeted therapeutic interventions for affected patients.

## 2. Purine Metabolism and Development of Dopaminergic Neurons

### 2.1. Dopaminergic Neuron Development

During embryonic development, the neural tube gives rise to most structures of the central nervous system, including the forebrain vesicle which is known as the prosencephalon [19]. The prosencephalon divides into the telencephalon (rostral), which will become the cerebral hemispheres, and the diencephalon (caudal) which will become the thalamus, hypothalamus, and optic vesicles [19,20]. The mesencephalon, the midbrain, arises from the mesencephalic vesicle and gives rise to the tectum, tegmentum, and cerebral peduncles [21].

One distributed set of neural formations (subcortical nuclei) throughout the telencephalon, diencephalon, and mesencephalon is the basal ganglia [19]. The basal ganglia are initially formed during embryogenesis but may not be fully developed until an individual is in their second decade, similar to the cerebral cortex [22].

**Figure 2:**
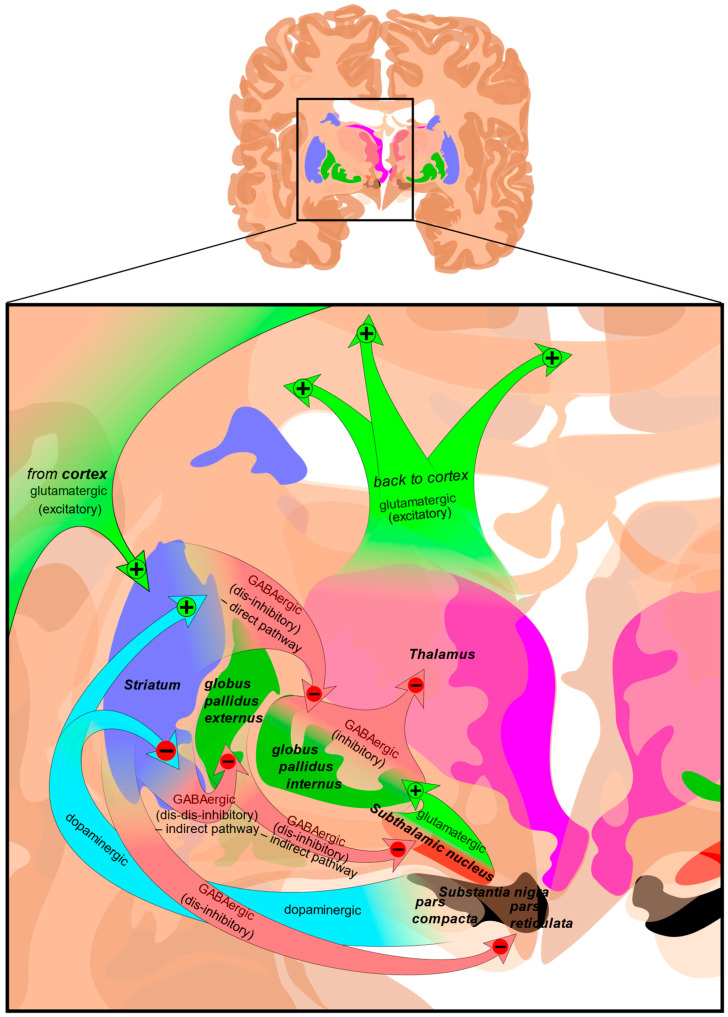
Structure and circuits of the basal ganglia. Two coronal slices have been superimposed to include the involved basal ganglia structures. + and − signs at the point of the arrows indicate, respectively, whether the pathway is excitatory or inhibitory. Green arrows refer to excitatory glutamatergic pathways, red arrows refer to inhibitory GABAergic pathways, and turquoise arrows refer to dopaminergic pathways [26].

The largest region of the basal ganglia is the striatum (Figure 2). The striatum is divided into two regions; the dorsal striatum contains the caudate and putamen, and the ventral striatum contains the nucleus accumbens, and ventromedial portions of the lenticular nuclei (the putamen, globus pallidus externus, and globus pallidus internus). Outside of the corpus striatum, the basal ganglia contain the substantia nigra which projects to the caudate nucleus and putamen in the dorsal striatum via the nigrostriatal pathway [23]. The ventral tegmental area and the retrorubal field are other dopaminergic nuclei originating in the midbrain which project to the prefrontal cortex via the mesocortical pathway (regulating cognitive function, motivation, and emotional response) and the ventral striatum via the mesolimbic pathway (regulating reward-based learning and addiction). The substantia nigra is further divided into the pars compacta which is dopaminergic and the pars reticulata which is GABAergic, both of which regulate motor control, reward learning, and are implicated in addiction and compulsive disorders [24,25].

**Figure 3:**
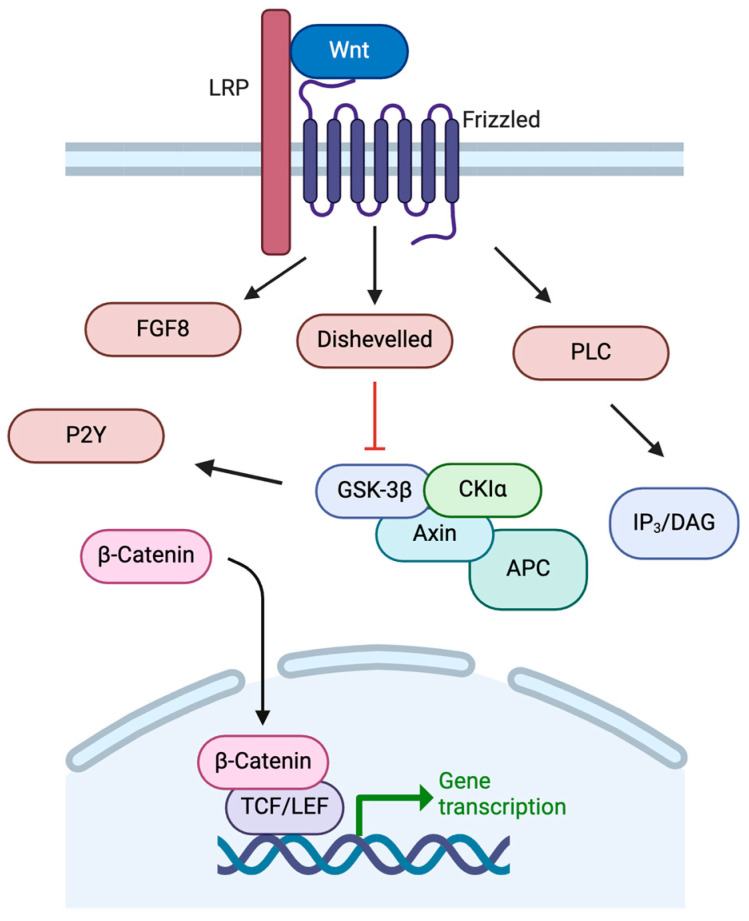
Wnt signaling pathways. See text for details. Created in BioRender. https://BioRender.com/m26o075 (accessed on 6 February 2025).

The development of the basal ganglia, including the dopaminergic neurons of the SNc, is controlled by a variety of temporal and regional protein expression patterns. One highly conserved family of signaling molecules, the Wnt proteins, is significantly involved in neural development [27]. Wnt proteins are synthesized and require modification via acetylation for secretion from the cell [28,29]. Wnt proteins bind to Frizzled receptors to activate multiple intracellular pathways in order to regulate embryonic patterning, neuronal differentiation, and neurogenesis (Figure 3) [27]. Once bound to Frizzled receptors, Wnt can activate pathways including the IP3/DAG pathway through PLC. Wnt can also inhibit GSK-3β, preventing degradation of β-catenin to allow its action as a nuclear transcription factor alongside TCF for genes such as c-MYC, fibronectin, and LMX1B, among others [27,30,31]. Activation of the Wnt/β-catenin pathway is crucial to dopaminergic cell fate.

Wnt/β-catenin signaling leads to FOXA2 expression in cells which can promote either dopaminergic cell expression or hindbrain serotonin cell expression [32]. In dopaminergic cells, FOXA1/2 can result in increased expression of metabolic enzymes relevant to the dopaminergic pathways including tyrosine hydroxylase (TH) and L-aromatic amino acid decarboxylase (AADC), thus allowing for dopamine release in the dorsal striatum.

There are several specific Wnt proteins that have been identified in dopaminergic neuronal development, including Wnt1 and Wnt4. Wnt4 is regulated by Prdm15 in embryonic development. Prdm15 (PR/SET domain 15) acts upstream of Wnt4 to regulate the development of the striatum from the lateral and medial ganglionic eminences [11,31,33]. Following embryonic day 11 (E11) in rat pups, Prdm15 no longer affects dopaminergic neuronal development, suggesting a very early role of Prdm15 [31].

Wnt1 is expressed in the embryonic midbrain to control dorsolateral midbrain patterning [34]. During embryonic neurologic development, Wnt1 stimulates FGF8 activity, which in turn forms the midbrain–hindbrain barrier (MHB), an essential boundary as the brain continues to differentiate [32]. Ventral midbrain dopaminergic neurons will have low expression of FGF8, whereas the hindbrain will a have high expression—this begins the differentiation process. The formation of the MHB results in the expression of SHH which leads to the activation of GLI, a transcription factor that inhibits Dkk1 (Wnt antagonist), Nkx2.2 (GABAergic transcription factor), and Nkx6.1 (controls fate of the red nucleus in the midbrain), and stimulates Ngn2 (for induction and differentiation of dopaminergic neurons) [32,35,36,37,38].

Additionally, the Wnt1/β-catenin pathway will stimulate Otx2, Lmx1a, Msx1, and Ngn2 [32]. Lmx1a inhibits Hes1, a protein associated with GABAergic neuronal fate, and is further stimulated through the Wnt/Lmx1a autoregulatory loop, thus encouraging dopaminergic cell differentiation. Wnt signaling also increases the activity of Engrailed-1 (En1). En1 can stimulate Ahd2, leading to retinoic acid (RA) production, as well as Cck [39,40]. RA is an essential vitamin needed in precise balance for embryonic development, especially that of the brain [41]. RA encourages the transcription of many factors, but in the SNc it can encourage production of tyrosine hydroxylase, which is essential for the development and maintenance of dopaminergic neurons [42]. RA is also a potent stimulator of GABAergic neurons in the striatum, potentially through different RA receptor isoforms [43,44].

### 2.2. Ectonuclease Expression

Alongside the increasing recognition of the importance of purinergic signaling in the developing brain, extracellular enzymes that hydrolyze nucleotides (ectonucleotidases) have also been recently investigated in the physiology of the CNS. Ectonucleotidases help regulate the tightly controlled levels of purine phosphates in the extracellular space. In the nervous system, particularly during development, this is a significant point of regulation for purinergic signaling [45,46].

The expression of several enzymes has been identified to increase during the postnatal time period including ectonucleotidases. Ectonucleotide pyrophosphatase/phosphodiesterase (E-NPP) has the capacity to catalyze hydrolysis of pyrophosphate or phosphodiester bonds including those in nucleotides and relevant CNS lipids such as choline phosphate esters [47,48]. E-NPPs hydrolyze ATP into AMP, which can be further hydrolyzed by membrane-bound ecto-5′-nucleotidases. NPP enzymes have been implicated in postnatal development, including NPP1 which shows age-related mRNA expression in both neurons and glial cells [49]. NPP1 expression has been investigated in regions such as the cerebral cortex, striatum, and hippocampus. NPP2 seems to serve a non-enzymatic function in early embryonic development, with a peak coinciding with NPP1 at PD7 throughout the brain. NPP3 expression is inversely related to NPP1 and 2 and decreases in similar areas at PD7 and through adulthood.

E-NTPDases1-3 (also known as CD39/L1/L3, respectively) cleave ATP to AMP and appear to be major regulators of purinergic P2 receptor activity and purine recycling [50]. Specifically, E-NTPDase3 expression peaks at PD7 and throughout adulthood in diencephalic and limbic structures, and can be found in striatal gray matter [49,51].

Finally, ecto-5′-nucleotidase (also known as CD73) catalyzes the formation of adenosine from AMP, which is the primary source of extracellular adenosine in rodent CNS development [49]. CD73 is linked to the membranes of neurons and glia throughout the brain, including in the caudoputamen, hippocampus, and cingulate cortex, also peaking around PD7. CD73 seems to be involved in crucial developmental processes such as migration, differentiation, and synaptogenesis through both enzymatic and non-enzymatic functions.

Overall, the concentration of extracellular purines and nucleotides is highly regulated, in part by ectonucleotidases. The concentration of these molecules during development is especially important for proper development, and changes in expression of ectonucleotidases play an essential role in these functions [52,53,54].

### 2.3. Energy Requirements

During postnatal neurological development, the brain is responsible for approximately 60% of the body’s basal energy requirements [55]. This high energy demand is particularly pronounced in dopaminergic neurons in the substantia nigra pars compacta (SNc), which are more vulnerable to damage under conditions of low energy (low ATP) compared to other neurons [56]. This vulnerability arises from their large, unmyelinated axonal arbors, which are substantially more complex than those of other dopaminergic neurons, making them more susceptible to energy deficits [1]. Microtubules are dynamic polymers comprising protein subunits that are essential for this complicated cellular structure and function. One protein subunit, tubulin, binds GTP directly and is required for microtubule polymerization [57]. Neurons that are highly arborized, such as the dopaminergic neurons of the nigrostriatum, would require substantial levels of GTP to develop the microtubules that help maintain this complicated cytostructure [58]. Disruptions to microtubule dynamics by a toxin, rotenone, showed a specific vulnerability of these dopaminergic neurons due to the impaired vesicle transport, leading to oxidation of dopamine and cell death [59]. Additionally, SNc neurons have high energy demands due to their autonomous pacemaking activity and broad action potentials, further increasing the need for purine synthesis to support brain function.

Purines, particularly adenosine and guanosine, are essential components of cellular energy. ATP and GTP serve as primary energy carriers in cells, powering various biochemical reactions and processes. ATP releases energy by hydrolyzing its phosphate bonds, fueling activities like muscle contraction, ion transport, and biosynthesis. Similarly, GTP provides energy and acts as a signaling molecule, especially critical in protein synthesis and signal transduction pathways. During embryonic development in rat models, de novo purine synthesis is the primary source of purines for the brain [60]. However, near birth, there is a transition in purine metabolism to the salvage pathway, particularly hypoxanthine-guanine phosphoribosyltransferase (HGPRT) activity, which becomes more prominent. HGPRT expression increases throughout the postnatal period, gradually replacing de novo synthesis for the salvage pathway as the dominant pathway for purine production.

ATP is most efficiently produced through oxidative phosphorylation in mitochondria. For this reason, mitochondrial dynamics also play a crucial role in meeting the energy demands of developing neurons. In younger neurons, mitochondria are smaller, more mobile, and occupy less space within neuronal processes compared to those of mature neurons [61]. This enhanced mitochondrial motility may allow for faster energy distribution in response to cellular demands. As neurons mature, mitochondrial mobility decreases, but the number of mitochondria per cell increases, indicating that neuronal differentiation requires an increase in mitochondrial number, potentially independent of ATP synthesis [61,62].

Interestingly, purine biosynthesis enzymes, such as phosphoribosylformylglycinamidine synthase (PFAS), phosphoribosyl aminoimidazole succinocarboxamide synthetase (PAICS), and the enzyme that catalyzes the last 2 steps of purine biosynthesis, 5-aminoimidazole-4-carboxamide ribonucleotide formyltransferase/IMP cyclohydrolase (ATIC), are not only distributed throughout the cell but also pool near mitochondria [63]. ATIC, in particular, has been found within mitochondria themselves, suggesting a direct link between purine metabolism and mitochondrial function in neurons [63,64].

Mitochondrial function is closely linked to nitric oxide (NO) signaling, as NO stimulates mitochondrial fission [65]. Mitochondrial fission is the division of mitochondria to maintain adequate supply and quality control, while fusion is the combination of mitochondrial gene products especially under cellular stress [66]. The balance of fission and fusion is essential for a high-quality and high-output production of ATP in the cell [67,68]. Additionally, maternal immune activation, which triggers the overproduction of reactive oxygen species (ROS) via NADPH oxidase (NOX), can result in mitochondrial damage that persists into infancy and prenatal ATP depletion [69]. This early mitochondrial damage may contribute to oxidative stress and neuroinflammation later in life, creating a self-perpetuating cycle of mitochondrial dysfunction and ROS generation.

### 2.4. Purinergic Signaling in the SNc During Development

Purines also act as neurotransmitters, both during development and into adulthood, and contribute to the regulation of dopaminergic neuronal function. Purinergic signaling occurs through purines binding extracellular receptors. Purinergic receptors fall into two families: P1 for adenosine signaling and P2 for ATP/ADP signaling [70,71]. There are four subtypes of P1 receptors (ADORA1, ADORA2A, ADORA2B, ADORAA3) and two P2 receptor subtypes (P2X and P2Y) [72]. Purinergic receptors are expressed in almost all tissues, but vary in isotype expression [73].

Dynamic expression of these receptors appears to play an important role in neural development. P2YRs are expressed as early as embryonic day 11 (E11) [74]. Specifically, the floor plate of the neural tube expresses P2Y1. At embryonic day 14, P2Y4 is the only purinergic receptor expressed in the brain, but by day 18 P2Y1 is expressed. Postnatally, P2Y4 receptor expression disappears from the midbrain, isthmus, and medulla, but this receptor is still present in the amygdala and diencephalon [74]. Notably, only the cortical amygdaloid nucleus expressed P2Y4.

P1 receptors are involved in many processes, including regulating energy balance, sleep, torpor, and hibernation. The four subtypes of P1 receptors each have a distinct but overlapping function [75,76]. ADORA1 receptors are largely located presynaptically, while ADORA2A receptors are predominantly found on somatodendritic regions. ADORA2A receptors are present in the striatum at birth and have similar properties as in adults [74]. Prior to 25 days of age in rats, the binding of adenosine to ADORA1 is 20% of adult levels. By 25 days, the adenosine has the same affinity for ADORA1 as that of adult rats. Xanthine modulates postnatal development of the basal ganglia through ADORA2A. ADORA2A acts through β-arrestin signaling to activate the striatal indirect pathway via medium spiny neurons (iMSNs) [77].

Dopaminergic receptors, D1R and D2R, form receptor mosaics with adenosine receptors throughout the basal ganglia [78]. RMs lend themselves to emergent behavior dependent on complex interactions between several receptors. There are patterns in D1R/D2R expression and purinergic receptor (ADORA2R) expression in the basal ganglia; however, these receptors are not necessarily co-localized, despite occurring on the same neurons [79]. Generally, ADORA1, ADORA2A, D1R, and D2R all increase postnatally in the basal ganglia, caudate–putamen, and other brain regions in the first weeks of life. Adult numbers of D1R and D2R are established by the end of the first postnatal month. Some refining occurs, and D1R seems to be lost more rapidly in the mature animal than D2R.

In adulthood, ADORA2AR in striatopallidal neurons mediates goal-directed behavior by enhancing cognitive flexibility through both positive and negative regulation of several sites [80]. These neurons integrate dopamine and adenosine signaling through multimeric ADORA2AR-D2R heterocomplexes to fine-tune local signaling changes. Interestingly, ADORA2AR antagonism has been found to prevent cell death associated with alpha-synuclein (a protein highly associated with Parkinson’s Disease) aggregation, potentially through an NMDA receptor-dependent mechanism [81,82]. This mechanism is exploited by the drug istradefylline, which is a known ADORA2AR antagonist used in conjunction with other drugs for management of Parkinson’s disease [83,84].

Glia, a group of non-neuronal support cells in the CNS, also contribute to SNc development through purinergic signaling. One type of glia, astrocytes, are heavily involved in the development and circuitry of the basal ganglia, particularly in the formation and maintenance of synapses [85,86]. Astrocytes are primarily formed postnatally but continue to change over time alongside neurogenesis and synaptic pruning in early life [87,88,89].

In astrocytes, P2 receptors are coupled to GSK3β by a PKC-dependent pathway following ATP binding [90]. The subsequent phosphorylation inhibits GSK3β, which can lead to changes in Wnt/β-catenin pathways in astrocytes [90,91]. Microglia, another subset of glia cells, contain P2Y12 which are activated by ADP with a neuroprotective function [92]. Microglia can also release nitric oxide and reactive oxygen species to degrade impaired dopaminergic neurons of the basal ganglia, potentially playing a role in conditions like Parkinson’s disease [93].

## 3. Uric Acid and the Development of Dopaminergic Neurons

### 3.1. Uric Acid

Uric acid (UA) is the final product of purine catabolism in humans. Healthy serum uric acid (SUA) levels are estimated at 5–7 mg/dL (0.2–0.43 mM) for men and 3–6 mg/dL (0.14–0.36 mM) for women, but the exact range for what is considered healthy varies greatly [94,95]. The concentration of SUA in humans is near the solubility point (7.0 mg/dL in plasma at 37 °C) [95]. This high concentration lends itself to UA precipitation and crystallization, which can lead to conditions like gout.

The high concentration of uric acid is maintained by heavy renal reabsorption and a lack of the enzyme uricase, which is present in other organisms to break down uric acid into a more soluble product, allantoin [96,97]. Hyperuricemia can lead to conditions such as gout and elevated uric acid in urine can lead to nephrolithiasis [98,99]. However, the development of disease is not directly proportional to uric acid concentration, because of the variable involvement of the innate immune system. Over time, elevated uric acid is likely to activate inflammation, thus contributing to the development of disease [100].

Uric acid is primarily excreted renally (60–70%), with the rest undergoing intestinal uricolysis [96]. In the kidneys, most circulating urate is freely filtered with roughly 90% of filtrate reabsorbed later by transporters including URAT1 and GLUT9. In the large intestine, resident gut bacteria containing uricase can break down uric acid into allantoin to be excreted in feces [96,101]. Allantoin can also be formed from the spontaneous oxidation of uric acid, typically in alkaline conditions or high oxidative stress [102]. Standard physiologic levels of allantoin range from 2.77 μM in men and 2.18 μM in women in serum, which are roughly 1000-fold lower than the concentration of uric acid [95,102].

During development, the fetus is exposed to uric acid. The placenta maintains uric acid homeostasis in pregnancy and in the fetal environment through GLUT9, a high-capacity uric acid transporter [103]. GLUT9 has two isoforms, GLUT9a (predominantly on basolateral surfaces) and GLUT9b (predominantly expressed on apical surfaces) [104]. GLUT9a and GLUT9b have been identified in the villous (apical) membrane of the placenta but not on the basal membrane of the syncytiotrophoblast [103,105]. Maternal levels of UA are closely correlated with fetal levels.

Hyperuricemia is present prenatally and postnatally in several clinical circumstances. During early pre-eclamptic pregnancies, for example, hyperuricemia is a common finding, often associated with low birth weight [106,107]. In pregnancies with hyperuricemia but without preeclampsia, lower birth weights were still observed when hyperuricemia persisted for more than 2 weeks [108]. Similarly, in a GLUT9 murine knockout (KO) model, neonatal mice lacking GLUT9 exhibited significantly lower birth weights and impaired renal development, as characterized by epithelial necrosis, attributed to the coincident hyperuricemia [109]. Placental System A amino acid transport, an active sodium-dependent transport system for neutral, short side chain amino acids, is critical for proper intrauterine growth [110]. Uric acid inhibits this transporter in a concentration-dependent manner, which could explain a link between fetal growth restriction and hyperuricemic preeclampsia. One study found increased GLUT9 placental expression during insulin treatment of pregnant individuals with diabetes, which is often associated with an increased birth weight [111,112,113].

During normal pregnancy, uric acid levels in pregnant women decrease significantly by 8 weeks gestation and remain below pre-pregnancy levels until 24 weeks gestation [114]. The mean values of non-pregnant women were 0.25 mM, and then by 8 weeks, levels decreased to 0.070 mM, with the lowest levels occurring at 12 weeks, measuring 0.047 mM. Increased maternal renal blood flow and uric acid excretion help to explain the decrease in uric acid levels during mid-pregnancy [115].

### 3.2. Uric Acid in the Brain

Uric acid is present in CSF; however, in healthy, physiologic conditions, the brain does not favor purine catabolism [116]. In developing human tissue, the primary catabolic enzyme that forms uric acid, xanthine oxidoreductase, has very low gene expression. In brain tissue specifically, only low levels of xanthine oxidase—one form of xanthine oxidoreductase—transcripts can be detected, explaining the lower levels of uric acid in CSF compared to serum.

The precursors to uric acid, xanthine and hypoxanthine, also exist in CSF and serum. Hypoxanthine levels in serum are around 0.56 μM [117,118]. In adults, CSF levels measure 1.8 μM of hypoxanthine and 1.7 μM of xanthine [118]. In infants, CSF hypoxanthine levels have a mean of 3.6 μM, while CSF xanthine has a mean of 5.0 μM, which are comparatively higher than adult levels.

Uric acid is mostly present as the charged anionic urate in serum; therefore, it cannot easily diffuse across membranes. However, there does appear to be a correlation between serum and CSF UA levels. In one study, serum biomarkers (including uric acid) were measured in individuals with Alzheimer’s disease (AD) [119]. While the study did not identify a correlation between serum UA and AD, it found that serum uric acid did influence CSF uric acid. On average, CSF UA was about 10-fold lower than plasma levels, and a 1 μM increase in plasma UA was associated with a roughly 5% increase in CSF UA. In individuals with blood–brain barrier (BBB) impairment (indicted via CSF Albumin Index of 9.0 or greater), CSF UA was 6.2 μM higher than controls.

Potentially, the relationship of serum UA to CSF UA levels may occur through the conversion and transport of hypoxanthine as a bidirectional hypoxanthine transport system through the blood–brain barrier [120]. Once hypoxanthine is taken up by this system, the brain quickly uses hypoxanthine for salvage by HGPRT to reform adenosine and guanosine. Additionally, high concentrations of hypoxanthine are able to leave the CSF and enter serum. The hypoxanthine transport system may explain the transport of purine stores from the liver; however, hypoxanthine uptake from circulation is most likely not the primary source of purines for the brain compared to purine salvage pathways and nucleoside uptake through transporters [120,121].

Unsalvaged hypoxanthine is converted to xanthine and oxidized to uric acid by xanthine oxidase, which uses molecular oxygen and produces hydrogen peroxide [16,122]. To maintain lower CSF UA levels, it is possible that hypoxanthine is excreted from the central nervous system into serum and then converted to uric acid by xanthine oxidase. Xanthine oxidase is expressed mostly in the liver, intestines, and vascular endothelial cells [16,116,123].

### 3.3. Antioxidant Functions

Unlike many other mammals, humans are unable to catabolize uric acid or synthesize ascorbic acid as they are double knockouts of the uricase gene and the L-gulonolactone oxidase (GLO) gene (an enzyme that catalyzes the last step of ascorbic acid synthesis). One theory suggests that uric acid assumed the antioxidant role of ascorbic acid following an inactivating mutation in the gene coding for GLO around 40 million years ago [124]. Ascorbic acid is the primary antioxidant in most mammals, while uric acid is suggested to provide most of the antioxidant capacity in human blood [125,126]. Uric acid may also improve ascorbic acid levels in humans by increasing its stabilization and, thus, availability [127]. Despite the physiologic shift in antioxidants, the double knockout resulting in an inability to synthesize ascorbic acid and to catabolize uric acid in humans is estimated to have occurred at different time scales, suggesting that the mutations are largely uncorrelated [128].

The antioxidant effect of uric acid seems to vary across anatomical regions, with some studies finding substantial neuroprotective functions in lipid peroxidation and intestinal H_2_O_2_-induced oxidative damage [129,130,131]. In contrast, uric acid may also induce oxidative stress intracellularly [129]. Oxidative stress impairs mitochondrial function which can affect organs, such as the brain, that are particularly susceptible to oxidative damage and the generation of superoxide radicals [132]. It is possible that uric acid has a physiologic function in the CSF as an antioxidant to prevent this damage [119].

The most robust antioxidant function of uric acid seems to be its ability to scavenge peroxynitrite. In the mitochondria, superoxide anions can be produced by the electron transport chain and can react with NO to yield peroxynitrite [133]. Typically, NO inhibits respiration, but this effect can be prevented by uric acid. Uric acid has potential neuroprotective effects through its neutralization of peroxynitrate, with UA supplementation being considered for improving ischemic injury [134].

The scavenging of peroxynitrite by uric acid leads to reactive intermediates, ultimately forming triuret as the final product in aqueous buffers [135]. Interestingly, ascorbic acid can partially prevent this reaction. Very little triuret is produced in healthy individuals but it has been identified in the urine of preeclamptic pregnant individuals. Triuret can spontaneously fragment into several different compounds, potentially explaining the pro-oxidant effects of uric acid on lipids and sulfhydryls [136].

Though UA is a potent radical scavenger in serum, its effect is limited, especially in hydrophobic environments. For example, it has been demonstrated that UA cannot reduce superoxide or act as an antioxidant in low density lipoprotein (LDL) molecules and cell membranes [129]. Notably, UA can degrade into several harmful forms, including carbon-centered radicals that predominantly target lipids in LDL and membranes.

Though the antioxidant effect of uric acid is complicated, its neutralization of peroxynitrite and its potential to perform other protective functions in lipid peroxidation may explain a physiologic role of uric acid beyond serving as just a purine catabolism product, particularly in the brain.

### 3.4. Pro-Inflammatory Functions of Uric Acid

Not only are high levels of uric acid associated with gout because of its limited solubility, but uric acid also plays a role activating inflammatory processes that contribute to disease. In fact, uric acid is involved in several molecular pathways relating to the immune system [137]. For example, through the ubiquitous NADPH oxidase-dependent pathway, UA itself can activate a pro-inflammatory state and increase oxidative stress.

Uric acid is sensed by TLR2 and TLR4, two types of pattern-recognition receptors (PRR). Their binding by uric acid leads to dendritic cell activation, contributing to the innate immune response [138,139]. Additionally, uric acid can trigger caspase-1 activation, which leads to IL-1β processing and maturation in a manner that is independent of TLR signaling but requires NALP3, an innate immune receptor, specifically in macrophages [138,139,140]. Given its association with cellular injury and its role in activating dendritic cells, uric acid appears to function as an endogenous signal of cell death [141].

Activation of PRR including those stimulated by uric acid can cause developmental changes in the brain. For example, maternal immune activation and the subsequent expression of pro-inflammatory cytokines (IL1β, TNF-α, IL-6) in the fetal brain, through TLR2 and TLR4 activation, are associated with morphological changes in the postnatal amygdala [142]. These changes include long-term microglial activation, mild astrogliosis, and the upregulation of TLR2 and TLR4, which persist in the postnatal period. The activation of microglia results in secretion of pro-inflammatory cytokines such as TNF-α, IL1β, and insulin-like growth factor 1, which positively regulate dopaminergic neurogenesis, reducing cell proliferation, apoptosis, and necrosis consistently across cell lines [143,144]. Furthermore, for example, following exposure to paraquat (a herbicide), microglial activation precedes dopaminergic cell loss in the substantia nigra by two weeks, involving increased CD86 expression, decreased CD206 expression, elevated levels of TNF-α and IL-6, and reduced levels of anti-inflammatory cytokines IL-10 and TGF-beta [145,146].

Prenatal maternal inflammation has been associated with effects on neurologic development, though the relationship with hyperuricemia is complicated. Uric acid’s activation of pattern recognition receptors TLR2 and TLR4, particularly in microglia that guide dopaminergic neuron development, may have a particular effect on substantia nigra function in postnatal life.

### 3.5. Uric Acid and Neurologic Disease

One disease consistently associated with altered uric acid levels is Parkinson’s disease (PD). PD is a primary motor disease largely associated with a loss of dopaminergic neurons in the substantia nigra [147,148]. In a comparison of individuals with PD, those with cognitive impairments showed lower serum uric acid levels than those without [149]. Generally, higher levels of uric acid are associated with a decrease in striatal neuron loss and better clinical outcomes [150,151,152]. This association is consistent across epidemiological and clinical studies; however, causation or reverse causation is still unclear [153].

## 4. Lesch–Nyhan Disease

### 4.1. Overview

The effect of purine metabolism on brain development is exemplified by Lesch–Nyhan disease (LND), a severe X-linked recessive deficiency in the HGPRT enzyme. As mentioned previously, HGPRT is a key enzyme involved in purine recycling, with a reciprocal regulatory relationship with phosphoribosyl pyrophosphate (PRPP), involved in the de novo synthesis of purines. LND results in selective changes in the basal ganglia, particularly in the dopaminergic neurons of the substantia nigra pars compacta, thus exemplifying the developmental impact of purine metabolism on these neurons.

Biochemically, HGPRT deficiency results in decreased purine recycling, which leads to increased purine degradation. This increased degradation is the basis for peripheral hyperuricemia found in patients with LND. Additionally, there is increased activity of the de novo synthesis pathway to compensate for the loss of salvage. Generally, the compensation of the de novo synthesis pathway is sufficient for purine synthesis, and relevant enzymes are compartmentalized into the purinosome near the mitochondria [154,155].

In these patients, hyperuricemia is typically present at birth, which results in the early manifestation of crystalluria, nephrolithiasis, and gout [156]. Orange crystals present in a child’s diaper are typically the first indications of LND, followed by delayed psychomotor development observed around 3 to 6 months. The motor symptoms of LND also resemble other disorders such as cerebral palsy. Notably, LND is often misdiagnosed as cerebral palsy due to similar age of onset and motor symptoms, including slow, writhing movement (athetosis) and involuntary muscle contractions (dystonia) [157,158,159]. Other neurologic symptoms, including self-mutilation and cognitive impairment, gradually become evident within the first years of life [160]. The prognosis of LND is poor, with patients rarely surviving into their third decade, typically due to respiratory infection, renal failure, or sudden unexpected death [156].

Despite peripheral hyperuricemia, accumulation of uric acid is not likely to be responsible for the neurologic symptoms of LND. In fact, CSF UA levels are often normal in patients due to low xanthine oxidase levels in the brain [161,162]. Among the variations in severity of HGPRT deficiency, nearly all forms have hyperuricemia, but only the most severe deficiencies result in psychomotor delay and self-mutilation. While the relationship between HGPRT deficiency, hyperuricemia, and systemic inflammatory manifestations are biochemically straightforward, the causes of neurologic symptoms of LND are largely unknown.

### 4.2. Existing Hypotheses of Neurologic Consequences of Lesch–Nyhan Disease

#### 4.2.1. Hypoxanthine Excess

Due to decreased xanthine oxidase activity in the brain, there is relatively normal levels of uric acid, but excessive levels of hypoxanthine and other oxypurines in the CSF. The levels of hypoxanthine in the CSF in individuals with LND are four times higher than controls, and higher than serum levels in the same patient [163]. Therefore, it has been hypothesized that high levels of hypoxanthine contribute to the neurological deficits associated with LND.

In cell culture studies, cells treated with excess hypoxanthine exhibited changes in essential dopaminergic developmental proteins [164,165]. Prior to differentiation, there was an observed increase in Wnt4, Wnt11, and LMX1B expression. After differentiation, the expression of En1 and tyrosine hydroxylase increase, while elevated Wnt4 expression persists [164,165]. However, the increase in Wnt11 and LMX1B is not maintained post-differentiation, suggesting that the effects of elevated hypoxanthine are only observed prior to differentiation. However, these results are in conflict with findings from post-mortem human analyses, which demonstrated a decrease in tyrosine hydroxylase and other dopaminergic enzymes, suggesting that alterations in dopamine metabolism may be independent of the effects of elevated extracellular hypoxanthine [165,166].

On the other hand, upregulation of various receptors involved in neurotransmission due to elevated hypoxanthine is consistent with post-mortem findings in LND [167]. Particularly noteworthy is that the receptors involved in dopamine signaling (DRD1), serotonin (5-HT7), and adenosine (ADORA2A/2B) are affected by hypoxanthine in a developmentally time-dependent manner [164]. Specifically, DRD1, ADORA2A, and ADORA2B are elevated prior to differentiation, with DRD1 and ADORA2A levels remaining elevated post-differentiation. 5-HTR7 levels increase only after differentiation, while no changes were noted in 5-HTR2A expression.

In summary, hypoxanthine seems to have its own effects on cellular function, potentially explaining some of the neurologic symptoms seen in LND. The changes in Wnt signaling seen in cell culture are especially concerning, given the essential role of Wnt during development. Additionally, the changes in adenosine and dopamine receptor expression may affect both development and lifelong neurologic function. However, the effects of excess hypoxanthine should be delineated from the effects of HGPRT deficiency, as they do seem to differ. Potentially, treatments that lower hypoxanthine may improve outcomes, but further research is needed.

#### 4.2.2. Tetrahydrobiopterin and Dopamine Deficiency

**Figure 4:**
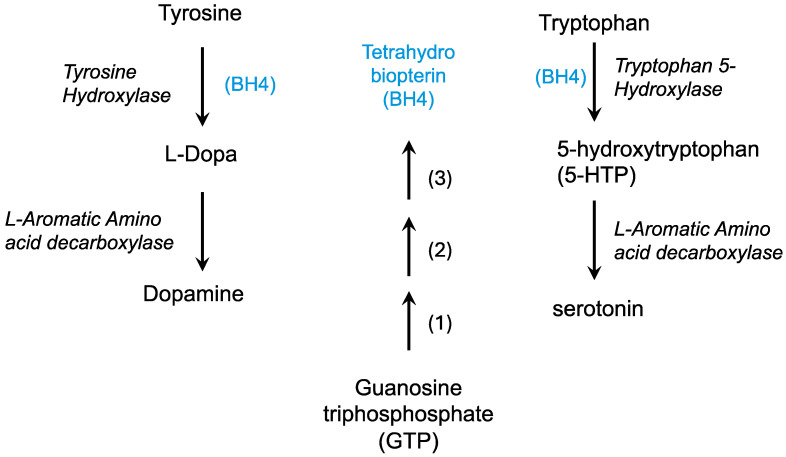
The formation of neurotransmitters requires tetrahydrobiopterin (BH4). Tetrahydrobiopterin (blue) is formed from GTP in three enzymatic steps: 1, GTP cyclohydrolase; 2, 6-pyruvoyltetrahydropterin synthase; 3, Sepiapterin reductase.

The dopamine deficiency theory in Lesch–Nyhan syndrome has been proposed based on biochemical deficiencies in dopamine, its catabolic product homovanillic acid (HVA), and changes in dopaminergic enzyme activity [168,169]. In dopaminergic neurons, tyrosine is hydroxylated by tyrosine hydroxylase using tetrahydrobiopterin (BH4) to form DOPA, which is then decarboxylated to form dopamine (Figure 4). Tyrosinase can convert DOPA to dopaquinone which can then become melanins, forming pigment in the skin and in dopaminergic regions of the brain.

Studies of postmortem brains from LND patients have identified decreased tyrosine hydroxylase (TH) staining in regions such as the midbrain, putamen, and substantia nigra although this finding is not always consistent [167,170]. Even in cases of reduced TH staining in the substantia nigra, some cells in this region appear to retain normal staining [166]. Additionally, a reduction in neuromelanization of these regions was observed, likely a downstream consequence of impaired dopamine synthesis, as decreased aromatic L-amino acid decarboxylase (AADC) activity has also been noted [165,166]. Studies in HGPRT knockout cell cultures also show reductions in both dopamine and its metabolites (DOPAC and HVA) [171].

A biochemical explanation for this lowered dopamine suggests that mutations in the HGPRT gene lead to a deficiency in GMP. As GTP is the substrate for de novo GTP cyclohydrolase I, this could disrupt the de novo synthesis of tetrahydrobiopterin (BH4), a necessary cofactor for TH activity [172]. This impairment may lead to reduced dopamine production.

Consistent with purine deficiency is the strong reduction in the purine sensor RHEB in dopaminergic neurons [171]. During purine shortage, adenylate levels and the TSC complex regulate GAP activity, leading to RHEB downregulation, and in chronic deprivation, RHEB degradation [173]. This deficiency is not observed in cortical cells or other induced pluripotent stem cells (iPSCs), suggesting significant specificity of purine depletion in SNc cells [171].

Furthermore, de novo purine synthesis governs mTORC1/SGK/S6 signaling, and purine nucleotide deficiency inhibits mTORC1 activity [173]. This signaling pathway is critical, as mTORC1 phosphorylation of pS6K stimulates translation by modifying the ribosomal subunit [60]. It is possible that a deficiency in purines could affect this pathway, leading to decreased protein translation that could explain the developmental changes observed.

Clinically, the motor symptoms of LND resemble those of Parkinson’s Disease, another condition associated with dopamine deficiency in the substantia nigra. However, unlike PD, Levodopa treatment in LND patients has proven ineffective and, in some cases, worsened both motor and affective symptoms [168]. This ineffectiveness may be linked to reduced levels of downstream enzymes, such as AADC, required for Levodopa’s conversion to the active molecule, dopamine [165]. Due to the developmental nature of LND, this observation may also be attributed to permanent changes in the SNc that are no longer able to be ameliorated by dopamine supplementation. It is important to note that gross changes in cell number, morphology, and density among dopaminergic neurons in LND are inconsistent, with most studies reporting no significant alterations [166,174].

Overall, dopaminergic neurons in the SNc seem to be the most affected by HGPRT deficiency, but the exact mechanism remains unclear. A tetrahydrobiopterin deficiency may explain the decreased dopaminergic synthesis, and if so, supplementation may improve patient outcomes. Maternal tetrahydrobiopterin supplementation would be ideal for proper fetal cognitive development if detected early enough, as BH4 can cross the placenta [175]. However, supplementation following early postnatal diagnosis may also help prevent onset of severe symptoms.

#### 4.2.3. Changes in Neural Connectivity During Development

Individuals with classic LND exhibit significant reductions in both white matter (26%) and gray matter (17%) compared to healthy controls [176]. These reductions are most pronounced in the medial inferior and frontal white matter regions, particularly those connecting the limbic, temporal, and motor cortex regions, aligning with the neurobehavioral characteristics of LND. Less severe variants of LND also show these patterns, though to a lesser degree.

Neuroimaging studies broadly suggest volume reductions in the brains of LND patients, sometimes reported as atrophy, though this pattern is not always consistent [154]. Given the developmental origin of these changes rather than a degenerative process, some researchers propose referring to them as dystrophy rather than atrophy. However, one case study documented atrophy in a patient over time, with a CT scan taken 8 years apart revealing volume loss, and postmortem analysis confirmed a loss of striatal neuropil (regions of gray matter containing neuronal and glial processes) potentially suggesting degeneration [85].

Further analysis indicates that the largest reductions in intracranial volume in individuals with LND are found in the basal ganglia, frontotemporal, and limbic regions, with relative sparing of the parieto-occipital regions [86]. Classic LND specifically shows reductions in the ventral striatum, prefrontal areas, temporal lobe, and left lateralized structures, whereas variant LND exhibits reductions in the lingual gyrus and precuneus, with sparing of frontotemporal regions.

At the cellular level, HGPRT knockout mice also show impaired proliferation and migration of midbrain dopamine neurons resulting in deviations in the migratory route and impaired dopaminergic circuitry [87]. This is associated with an abnormally structured radial glia-like scaffold, which may explain the disorganized innervation observed in the prefrontal cortex and decreased innervation in the primary motor and somatosensory cortices.

Obsessive-compulsive behaviors observed in LND may be linked to dysfunctions in the dopaminergic microcircuits of the substantia nigra pars compacta (SNc) and ventromedial striatum (VMS) [25]. The self-harm behavior in LND has often been characterized as compulsive like behaviors such as body-focused repetitive behaviors, potentially suggesting a mechanism similar to OCD.

Specifically, compulsive behaviors are associated with D1 receptor activation in the SNc-VMS pathway, while D2 receptor activity in the SNc-lateral orbitofrontal cortex pathway seems to inhibit these behaviors. This disruption in dopaminergic signaling is likely involved in the pathology of LND. Additionally, weaker projections from the substantia nigra to the putamen have been noted, which may contribute to motor and behavioral symptoms [166]. Upregulation in postsynaptic dopaminergic receptors have been noted in post-mortem studies [167].

#### 4.2.4. Changes in Energy Efficacy

In cell models of Lesch–Nyhan disease, reduced levels of AMP are evident before differentiation, while post-differentiation, lower levels of IMP occur [169]. All mutant models with a deficiency in HGPRT display excess hypoxanthine and xanthine, reflecting substantial purine loss due to the inactivity of the salvage pathway. Dopaminergic neurons exhibit increased concentrations of adenine and guanine metabolites, regardless of HGPRT activity, when compared to other cells in the central nervous system [171]. HGPRT knockout neurons show a disruption in both glycolysis and oxidative phosphorylation, without any observed structural changes to the mitochondria. Instead, glucose metabolism is shifted towards the pentose phosphate pathway, prioritizing nucleotide synthesis over energy production.

HGPRT deficiency may lead to diminished mitochondrial respiration in Complex I of the respiratory chain, which increases mitochondrial NADH levels and reactive oxygen species [177]. Additionally, HGPRT deficiency has been linked to elevated citrate levels and reduced lipid and fatty acid content [178]. It is also associated with decreased mitochondrial membrane potential [154,178].

Tetrahydrobiopterin is used in the synthesis of NO, which can drive fission of mitochondria [65]. Mitochondrial fission and fusion are typically maintained in balance, and changing that balance can lead to impaired cellular metabolism [65,68,179]. Given the tetrahydrobiopterin deficiency previously mentioned, it is possible that a lack of NO interferes with cellular respiration and metabolism.

Overall, complex changes in metabolism have been observed in dopaminergic neurons of the SNc in LND. Given the fragile nature and metabolic demands of these neurons, these effects could significantly contribute to the developmental impacts observed in LND.

#### 4.2.5. GPRT’s Direct Role in Dopaminergic Neuron Formation

HGPRT-deficient neuronal cell lines have elevated phosphodiesterase 10A (PDE10A) expression, disrupted cAMP/PKA signaling, and reduced cAMP response element-binding protein (CREB) levels [180]. This contrasts with in vivo studies in rats, which have shown significantly increased cAMP [178]. In HGPRT KO rats (which retain uricase), uric acid and hypoxanthine are wild-type, yet significant metabolomic changes are observed in nucleotide and general metabolism. Elevated citrate and orotidine levels were observed alongside reductions in lipids, including phospholipids and fatty acids. Additionally, increases in pyrimidine derivatives UMP and CMP, as well as cAMP and glucose-6-phosphate, were noted. Given the high energy requirements of developing neurons, disruptions to glucose utilization, the citric acid cycle, and purine supply would certainly disrupt proper function. Additionally, CREB phosphorylation is part of dopaminergic signaling, and impaired CREB signaling has been associated with SNc neuronal degeneration [181,182].

Other studies of stem cell models of LND show reduced FOXA1/2 and LMX1A expression in early development of induced pluripotent stem cells, as well as decreased OTX2 [171]. These models also exhibit reduced mTORC1 activity, leading to decreased ULK1 phosphorylation. Other models show upregulated expression of Sox2, a transcription factor that is not found in dopaminergic progenitors, and an increased expression of glia cell markers in neurons [183,184].

In HGPRT KO NT2 cells after differentiation, expression of LMX1A, MEX1, NGN2, FOXA1, and VMAT2 increased, while expression of Mash1, Nurr1, Pitx3, and AADC decreased [165]. Given the substantial impact of these proteins on dopaminergic neuronal development, it is possible that the changes in expression are relevant to the etiology of LND. It remains unclear what the exact mechanisms are by which changes in expression of individual genes impact development.

Purinergic signaling alterations in LND may also affect the development of dopaminergic neurons [91]. Decreased mRNA expression of P2Y1 and P2X3, along with increased NTPase expression, has been documented. P2Y1 expression, involved in ATP signaling and neuronal migration, was downregulated at both the expression and protein levels. This downregulation was accompanied by decreased CREB levels, but no change in ERK. Additionally, β-catenin phosphorylation was significantly decreased [91]. HGPRT KO cells had a marked reduction in total cellular β-catenin, providing evidence that HGPRT disrupts Wnt/β-catenin signaling.

The effect of HGPRT on essential developmental pathways warrants further investigation. Independent of hypoxanthine, HGPRT seems to directly influence the development of dopaminergic neurons and their postnatal function by regulating key proteins, such as FOXA, VMAT2, and CREB.

### 4.3. Therapeutic Implications and Future Directions

#### 4.3.1. Current Treatments for LND

The standard course of treatment for patients with Lesch–Nyhan disease primarily focuses on symptom management. For peripheral hyperuricemia, patients are typically prescribed allopurinol to block the conversion of hypoxanthine and xanthine to uric acid, decreasing serum uric acid. Hyperuricemia often leads to renal damage, kidney stones, and gout, but allopurinol is often able to treat these peripheral symptoms. However, there are instances where patients do not respond adequately to allopurinol treatment; in such cases, alternative treatments have been shown to be beneficial. Reducing serum uric acid levels in patients with LND does not ameliorate neurologic symptoms [185].

Currently, there is no standardized treatment for the neurological symptoms of LND, which can vary significantly among patients. Generally, medications such as baclofen or benzodiazepines, including diazepam, are utilized to address spastic motor symptoms. Additionally, benzodiazepines have been useful in mitigating behavioral symptoms such as anxiety. Other psychiatric medications that are commonly employed include Depakote, gabapentin, and carbamazepine. Risperidone, an antipsychotic, used in conjunction with S-adenosyl methionine, has also been found to help manage dystonia and self-injurious behavior [186].

Patients with LND often require physical restraints to prevent self-mutilation, typically applied to the hips, chest, and elbows. Strategies such as wrapping the hands and obscuring them from view may decrease the compulsive urge to self-harm. In severe cases, the use of mouth guards or tooth removal may be necessary to prevent lip and tongue biting.

#### 4.3.2. Potential for Early Levodopa Intervention

Although levodopa treatment has largely proven ineffective for LND patients, there is potential for early intervention to yield positive outcomes. One case study examined the administration of levodopa to infants aged 9 to 11 months who were diagnosed with LND [187]. Generally, disease severity can be predicted by HGPRT enzyme activity, with full gene knockout representing the most severe phenotypes [188,189]. Despite possessing alleles strongly associated with self-mutilating behaviors, these patients did not develop such symptoms [187]. This observation suggests that a time-sensitive treatment course may be viable for individuals with LND. Further investigation is warranted to explore early levodopa treatment regarding dosing and potential long-term outcomes. These results further suggest that interventions during critical postnatal neurological development periods may provide prophylactic treatment options for the developmental consequences of LND.

#### 4.3.3. Potential for Newborn Screening

Considering the critical periods of postnatal development and the deficiencies associated with LND, early detection and treatment options are essential for the prevention of symptoms and management of disease progression. It is generally accepted that individuals with LND exhibit hyperuricemia at birth; however, symptoms typically manifest only upon the observation of the presence of crystals in the urine. Therefore, implementing a routine uric acid serum test could facilitate the identification of LND prior to the onset of symptoms. Other causes of neonatal hyperuricemia, such as Down syndrome (trisomy 21), hypoxia, and maternal hyperuricemia, should be considered when ruling out alternative conditions in instances of hyperuricemia.

In addition to serum testing, a highly precise HGPRT enzyme assay can be used as a follow-up to idiopathic hyperuricemia to confirm a diagnosis of LND. This assay is expected to be more cost-effective than molecular genetic analysis and would offer a relative conclusive diagnosis. Given that symptom severity correlates with enzyme activity, this assay may serve as a useful indicator of potential disease outcomes. Molecular genetic analysis can ultimately confirm the diagnosis and further evaluate HGPRT activity for anticipatory care.

#### 4.3.4. Other Relevant Diseases of Purine Metabolism

Aside from Lesch–Nyhan syndrome, other mutations in purine metabolism can have profound impacts on the central nervous system, particularly the basal ganglia, manifesting in childhood diseases of motor and cognitive dysfunction. These diseases include ARTS syndrome and IMPDH deficiency.

ARTS syndrome is an X-linked disorder caused by a loss of function mutation in phosphoribosyl pyrophosphate synthase I (PRSI) that manifests with primarily neurologic symptoms including sensorineural hearing loss, intellectual disability, and delayed motor development [190,191]. PRSI catalyzes the first step in purine synthesis, the formation of phosphoribosyl pyrophosphate (PRPP). PRPP is also used in the purine salvage pathway. PRS isoforms are expressed in many tissues throughout the body, and PRSI, in particular, is found in especially high quantities in the brain and adrenal glands [192]. In a case study of two individuals with severe ARTS syndrome, nucleotide analysis in erythrocytes suggested a depletion in GTP, which could impact dopamine synthesis in the CNS [193]. This was accompanied by changes in white matter and other neuro-abnormalities. However, no physical changes were observed in the basal ganglia during neuroimaging.

Gain-of-function mutations have also been identified in the PRSI gene, resulting in increased purine production. Clinically, this manifests as childhood hyperuricemia, hyperuricosuria, and gout [191,194]. In severe forms, hypotonia, ataxia, and sensorineural hearing loss may be present [194,195].

Inosine monophosphate dehydrogenase (IMPDH) catalyzes the first step in the de novo biosynthesis of guanine nucleotides from inosine monophosphate. There are two isoforms of IMPDH, IMPDH1 and IMPDH2. IMPDH1 mutations seem to have selective ocular effects, whereas IMPDH2 mutations lead to childhood neuropathies with pronounced dystonia [196,197,198,199]. Both isoforms of IMPDH have been demonstrated to be ubiquitously expressed throughout adult and fetal tissues, with IMPHD2 being essential for life [200,201].

While further research is needed to demonstrate direct effects of PRS and IMPDH mutations on the basal ganglia, the developmental motor delays, dystonia, and other motor symptoms imply that the basal ganglia are directly impacted. It is interesting that despite these enzymes being expressed throughout the body and in different phases of development, mutations in these proteins can have highly selective effects on the central nervous system. This phenomenon emphasizes a critical role for purine metabolism and purine balance in neurodevelopment, with the basal ganglia appearing especially sensitive. Further research is needed to understand these fundamental roles and improve treatment of these poorly managed diseases.

## 5. Conclusions

**Figure 5:**
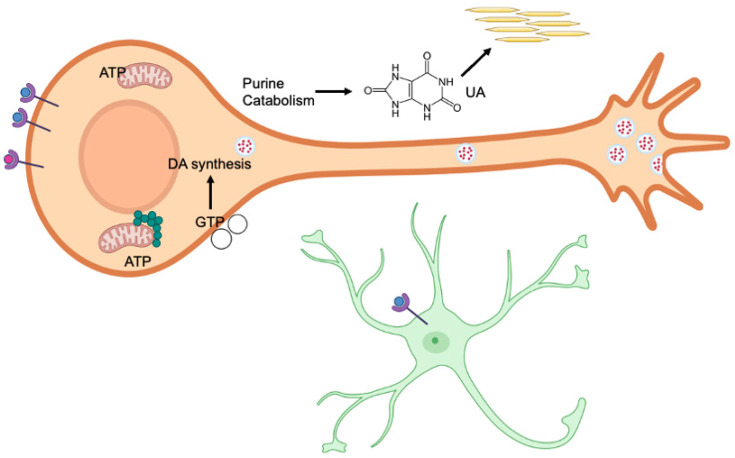
Purinergic receptors in the P1 subfamily are activated by binding to adenosine, while the P2 subfamily is activated by binding to nucleotides such as ATP (blue spheres). Receptors also bind dopamine (red spheres), which may interact with purinergic receptors. The dynamic expression of these receptors plays an important role in neural development. Generally, purinergic and dopamine receptors increase postnatally during the first weeks of life, thereby affecting neural development. Both embryonic and postnatal development require a supply of purines to support neurogenesis, synaptogenesis, and myelination. Purine synthesis primarily occurs in purinosomes (green circles), which are located near mitochondria. In contrast, the salvage pathway recycles free purine bases and nucleosides derived from nucleic acid breakdown. Disruptions in either pathway can cause significant biochemical imbalances, leading to anatomical, cellular, and biochemical changes. GTP is required for the synthesis of tetrahydrobiopterin, which in turn is needed for dopamine (DA) synthesis (red dots). GTP is also essential for the development of dopaminergic neurons, aiding in the polymerization of microtubules to create the complex structures of these cells. In astrocytes (green asterisk-like figure), ATP binding to P2 receptors leads to changes in the Wnt/β-catenin pathway, which is involved in synapse development. Ectonucleotidases in the extracellular space (open circles) decrease the levels of purine phosphates by hydrolysis. These enzymes peak in expression at specific time points shortly after birth and may contribute to neural development. The end product of purine catabolism is uric acid (UA), which acts as an antioxidant and, for example, can neutralize peroxynitrite (ONOO−). Excess uric acid may form crystals in the blood serum or kidneys.

Purine metabolism, relevant enzymes, and even uric acid, a metabolic waste product, might have substantial roles in neurological brain development, particularly that of dopaminergic neurons in the substantia nigra pars compacta (Figure 5). More research is needed to determine the specific and direct roles of purine metabolizing enzymes such as HGPRT in dopaminergic neuronal development, and how metabolic products such as hypoxanthine and uric acid may influence these functions (Table 1). These mechanisms can reveal crucial processes in neurologic development, as well as offer novel and effective treatment options for the developmental diseases such as Lesch–Nyhan disease.

## Figures and Tables

**Table 1:** Summary of diseases associated with purine metabolic enzymes.

Condition	Associated Enzyme	Comments	OMIM ^1^
Hyperuricemia and Gout	Xanthine oxidase	Inhibition reduces uric acid levels	607633
Hyperuricemia and Gout	Hypoxanthine Guanine Phosphoribosyltransferase (HGPRT)	Partial deficiency in enzyme function	308000
Lesch–Nyhan disease (LND)	Hypoxanthine Guanine Phosphoribosyltransferase (HGPRT)	Deficiency in enzyme function	308000
Hyperuricemia	GLUT9	Inhibition induces hyperuricemia	606142
Parkinson’s	ADORA2AR	Inhibition used to manage Parkinson’s	102776
ARTS syndrome	Phosphoribosylpyrophosphate synthetase I (PRSI)	Loss of activity	311850
Hyperuricemia	Phosphoribosylpyrophosphate synthetase I (PRSI)	Gain of activity	311850
IMPDH deficiency	Inosine-5′-monophosphate dehydrogenase (IMPDH)	Needed for synthesis of GTP	146690

^1^ Online Mendelian Inheritance in Man (OMIM) entry numbers to corresponding human genes and genetic phenotypes. Available online: https://omim.org/ (accessed on 6 February 2025).
